# The clinical spectrum of MELAS and associated disorders across ages: a retrospective cohort study

**DOI:** 10.3389/fneur.2023.1298569

**Published:** 2023-12-14

**Authors:** Benjamin C. Cox, Jennifer Y. Pearson, Jay Mandrekar, Ralitza H. Gavrilova

**Affiliations:** ^1^Department of Neurology, College of Medicine, Mayo Clinic, Rochester, MN, United States; ^2^Department of Health Sciences Research, College of Medicine, Mayo Clinic, Rochester, MN, United States

**Keywords:** MELAS, mitochondrial disease, stroke, genetics, epilepsy, myopathy

## Abstract

**Objective:**

Mitochondrial encephalomyopathy, lactic acidosis, and stroke-like episodes (MELAS) is a severe multisystemic disease, although some have a milder phenotype. We aimed to evaluate the clinical spectrum of this disease from MELAS patients to asymptomatic carriers and identify predictors of severity.

**Methods:**

We reviewed 81 patients, who had MELAS or had positive genetics without meeting clinical criteria. Patients who met criteria including lactic acidosis, encephalomyopathy, and stroke-like episodes (SLE) were categorized as MELAS, symptomatic non-MELAS, and asymptomatic. MELAS was further categorized as “standard-onset” if the first stroke-like episode (SLE) occurred before age 40 or “late-onset.”

**Results:**

Eighty-one patients were included: 42 MELAS (13 late-onset), 30 symptomatic non-MELAS, and 9 asymptomatic. MELAS patients had lower BMI at onset (mean 18.6 vs. 25.1 asymptomatic and 22.0 symptomatic non-MELAS, *p* < 0.05). There was a trend toward higher serum heteroplasmy in MELAS compared to symptomatic non-MELAS and asymptomatic (means 39.3, 29.3, and 21.8% *p* = 0.09). Symptomatic non-MELAS had more sensorineural hearing loss as first presenting symptom (51.6% vs. 24.4%, *p* < 0.05). MELAS had higher prevalence of seizures (88.1% vs. 16.7%, *p* < 0.05) and shorter survival from onset to death (50% mortality at 25 years vs. 10%, *p* < 0.05). Late-onset MELAS had longer disease duration from first symptom to first SLE (mean 16.6 vs. 9.3 yrs) and also lived longer (mean age at death 62 vs. 30). Standard-onset MELAS had more neurologic involvement at onset than late-onset (51.7% vs. 15.4%). Late-onset patients had more prevalent diabetes (69.2% vs. 13.8%) and nephropathy (53.8% vs. 10.3%). Patients with late-onset MELAS also had more organ systems involved (mean 4.1 vs. 2.7, *p* < 0.05). There was a trend toward higher heteroplasmy levels in standard-onset (mean 44.8% vs. 25.3%, *p* = 0.18).

**Discussion:**

Our study highlights the spectrum of MELAS. The lower BMI in MELAS at presentation as well as higher rates of sensorineural hearing loss as initial symptom in symptomatic non-MELAS may be useful clinical markers. While many patients present before age 40 with SLE, some can present with SLE later in life. Standard onset MELAS is more likely to present with neurologic symptoms. Late-onset is more likely to suffer diabetes or nephropathy and have more organ systems involved.

## 1 Introduction

Mitochondrial encephalomyopathy, lactic acidosis, and stroke-like episodes (MELAS) is a multisystemic disease characterized by the presence of stroke-like episodes (SLE). Although it typically presents with first SLE in adolescence to young adulthood and was originally defined as presenting with SLE before age 40 (1), cases of later onset of SLE are reported (2–4). MELAS affects multiple organ systems with nerve and muscle being most commonly affected, but also eye, skin, ear, liver, endocrine and heart (5). It is most commonly associated with MTTL1 m.3243A > G mutation in mitochondrial DNA (6), however, there are many individuals who carry this mutation who remain asymptomatic or do not meet clinical criteria for MELAS (7). In addition to the classic MTTL1 m.3243A > G mutation a variety of other mitochondrial DNA mutations have been associated with MELAS (8–11).

While the original diagnostic criteria outlined the features noted above, there has been a growing understanding of the phenotypic and genotypic characteristics of these disorders such that a recent revised diagnostic guideline was published (12). Proposed criteria include stroke-like episodes (sudden-onset focal neurological deficit with brain MRI or CT showing a cerebral lesion that does not conform to a large vessel territory and typically affects cortex and adjacent white matter) and encephalomyopathy, which includes clinical manifestation of skeletal muscle disease, encephalopathy (including cerebellar or sensory ataxia, chorea, dementia, seizures, strokes or stroke-like lesion, and Parkinsonism), and not fulfilling criteria for any other mitochondrial syndrome. The new criteria also highlight the importance of diagnostic testing, including biochemistry, genetic, histology, metabolic, and imaging.

The original diagnostic criteria of MELAS included the first stroke-like episode (SLE) presenting before age 40. As more has been learned about MELAS there has been greater appreciation for cases presenting much later in life with cases reported of patients with their first SLE presenting in their 40s to 60s (2–4, 13). A population-based study in Japan observed a bimodal distribution in the initial SLE presentation and saw that patients with juvenile MELAS (first SLE <18 years) had higher incidence of short stature in the juvenile form, whereas hearing loss, cortical blindness, and diabetes were more commonly seen in the adult-onset group (first SLE ≥18). There was also decreased survival in juvenile patients compared to adults.

Varying levels of heteroplasmy in different tissues is thought to be an explanation for the varying clinical phenotypes. Heteroplasmy levels have been shown to affect mitochondrial size and function (14). Variations in heteroplasmy levels may also be seen in different organ systems and also at different patient ages (15).

We aimed to evaluate the clinical spectrum of MELAS in a large North American cohort including patients presenting with MELAS throughout life, patients with MELAS-associated genetic mutations who have symptoms of mitochondrial disease but do not meet full MELAS criteria, and asymptomatic carriers. We aimed to identify clinical and genetic factors associated with more severe syndrome and/or late presentation of MELAS. We sought to elucidate clinical factors that could help differentiate patients who develop MELAS and to help identify which patients may present earlier or later with SLE.

## 2 Methods

We reviewed all Mayo Clinic patients seen in genomics clinic from January 1, 1996 (year of first clinical encounter for MELAS in our records system) through September 2021 for evaluation of mitochondrial disease. Our study was approved by our institutional review board under a waiver of informed consent. Our clinical database of patients with mitochondrial disease was reviewed for patients who met clinical criteria for MELAS as well as patients who had positive genetic testing for MELAS but did not meet the full clinical criteria. We categorized patients into 3 categories: MELAS (encephalomyopathy, lactic acidosis, and SLE), symptomatic non-MELAS (had positive genetic testing and symptoms of mitochondrial disease including myopathy, diabetes, sensorineural hearing loss, ophthalmoplegia, ataxia, cardiomyopathy, nephropathy, gastric dysmotility, etc. but did not meet full MELAS criteria), and asymptomatic patients (had positive genetic testing, due to the presence of MELAS in a family member, but did not have clinical signs of mitochondrial disease). Encephalomyopathy was determined by mental status exam and clinical and EMG and/or serologic evidence of muscle involvement. Stroke-like episodes were defined as acute focal neurologic deficits presenting with lactic acidosis and accompanied by typical cortical T2 changes on MRI with diffusion weighted restriction not respecting vascular territories (16).

Genetic mutations included the classic MTTL1 m.3243A > G, but also other mutations that have been associated with MELAS including MTTL1 m.3251A > G (17), MTTL1 3260A > G (11), MTND5m.13513G > A (18), MTND5m.13045A > C (18), MTTV1624C > T (9). In addition, for patients who were evaluated prior to widely available genetic testing, biopsy results from muscle or brain consistent with MELAS were considered confirmatory testing. Patients who met clinical criteria in the absence of confirmed genetic or pathologic testing were included if family history was supportive. Patients were excluded if their clinical phenotype and genetic testing was more consistent with an alternate diagnosis (e.g., POLG-1 or Leigh’s disease).

Patient charts were reviewed (authors BC and JP). Patient characteristics were extracted including age at symptom onset, age at diagnosis, duration of follow up, age at death (if applicable), and BMI at first presentation. Laboratory testing including pathology, genetic testing and heteroplasmy level (if present), mean baseline lactate and peak lactate level during SLE was reviewed. Clinical symptoms/signs were recorded including SLE, cardiac (including cardiomyopathy or arrhythmias), dementia, developmental delay, diabetes, nephropathy, gastric dysmotility, headaches, sensorineural hearing loss, ophthalmoplegia, seizures, myopathy (evidenced by exam, EMG, and/or muscle pathology), gait impairment, need for assistive device, and ataxia. The patient’s first presenting symptom was also recorded. Patient symptoms were further classified by organ systems (neurologic, cardiac, musculoskeletal, gastrointestinal, endocrine, etc.). If present, the age at first SLE and the age at death were recorded, as well cause of death, if known. Age at last follow up or age at death was also recorded. Radiographic imaging (CT, MRI, and/or MR Spectroscopy) was reviewed for characteristic changes of SLE as well as for chronic changes of cerebellar and/or global atrophy.

Patients who met criteria noted above were categorized as MELAS. Patients who did not meet full criteria, but who nevertheless had clinical symptoms of mitochondrial disease were categorized based on their symptoms. These categories included myopathy, maternally inherited diabetes and deafness (MIDD), deafness alone, mitochondrial encephalopathy without SLE (ME), and chronic progressive external ophthalmoplegia (CPEO). These categories were grouped together as “symptomatic non-MELAS.” Patients who did not exhibit any clinical features but who tested positive for mitochondrial DNA mutation associated with MELAS were labeled “asymptomatic.” Patients with MELAS were then grouped into “late onset,” which was defined as having first SLE after age 40, or “standard onset” in which SLE presented between ages 0 and 40. Age 40 was arbitrarily chosen based on prior classifications of MELAS as presenting before age 40 (1).

### 2.1 Statistics

Statistical analysis was performed (authors BC and JM) using SASS and BlueSky Statistics software v. 7.10 (BlueSky Statistics LLC, Chicago, IL, USA). Categorical variables were compared using Fisher’s exact test. Continuous variables were compared using univariate analysis. Kaplan Meier survival curves were analyzed using Log-Rank method. Unless otherwise stated, value of *p* of <0.05 was considered significant.

### 2.2 Standard protocol approvals, registrations, and patients consents

The study was approved by the institutional review board of Mayo Clinic (Rochester, MN), and all patients consented to the use of their medical records for research purposes.

### 2.3 Data availability

Data not provided in the article because of space limitations may be anonymously shared at the reasonable request of any qualified investigator for purposes of replicating procedures and results.

## 3 Results

### 3.1 Patient demographics

**Figure 1:**
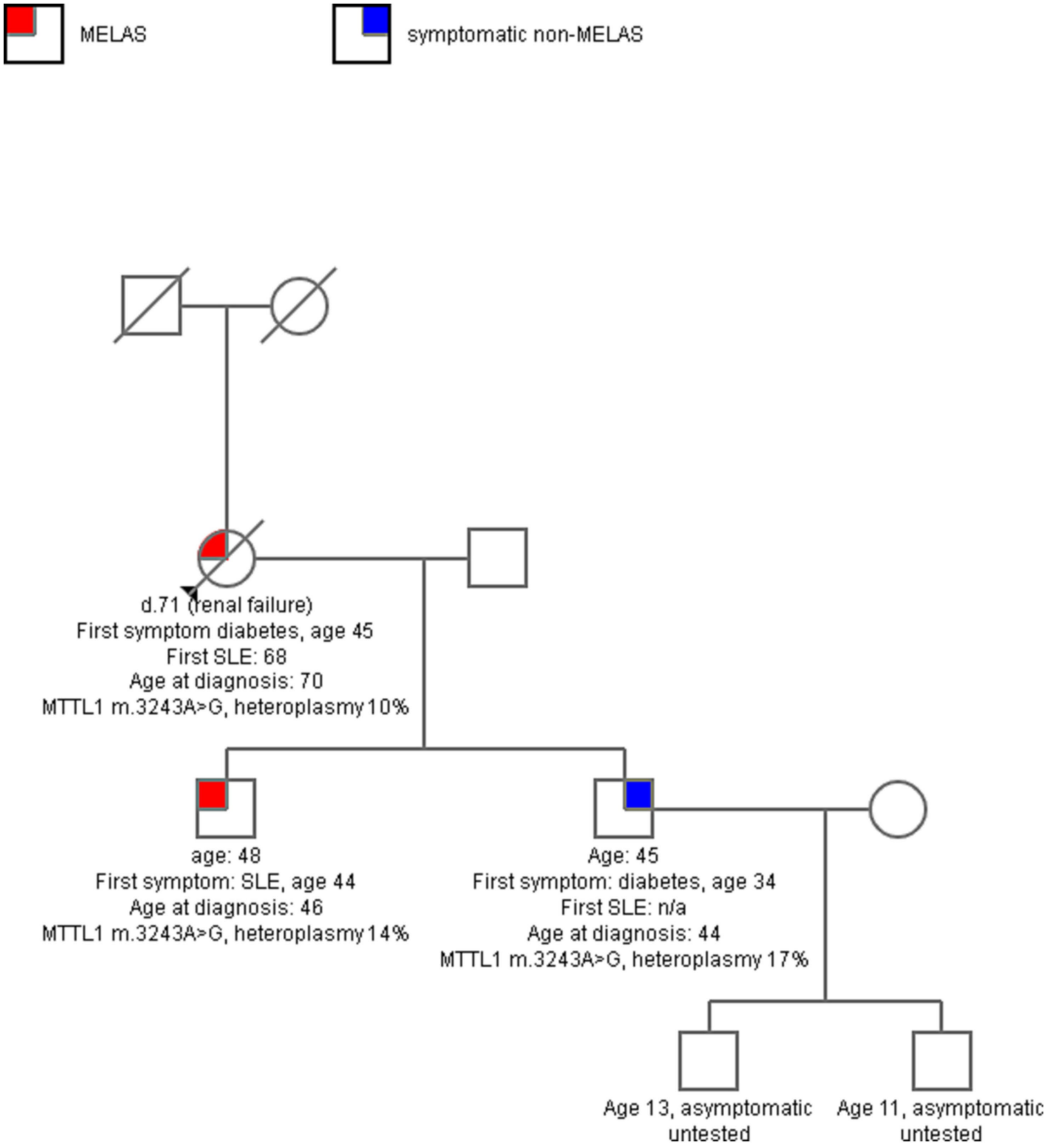
A sample pedigree of one family with 3 affected, two with MELAS and one with maternally-inherited diabetes and deafness (MIDD). The mother had longstanding diabetes, sensorineural hearing loss, and then developed progressive ataxia in her 60s, with multiple SLE in late 60s. Both sons in their 40s had symptoms of mitochondrial disease, but one had more severe disease with multiple SLE and seizures and the other had milder MIDD. Serum heteroplasmy level of MTTL1 m. 3243A > G was comparable across all three.

Eighty-one patients met inclusion criteria, with mean follow up of 5.8 years (SD 4.7). Twenty patients had another family member in the cohort with a total 8 families represented without consanguinity (see Figure 1 for example pedigree). The remaining 60 patients were unrelated. Twenty-two patients in the cohort died and dementia was the leading cause of death (5 patients), followed by unknown (4), seizure (3), renal failure (2), bowel obstruction (2), stroke (2), heart failure (2), non-small cell lung cancer (1), and multisystem organ failure. Of note, neurologic causes of death (dementia, stroke, seizures) accounted for 45% of the deaths. Of the total cohort, 42 patients met criteria for MELAS, 30 were symptomatic non-MELAS, and 9 patients were asymptomatic. Of the symptomatic non-MELAS patients, 16 had MIDD, 6 had ME, 5 had myopathy, 2 CPEO, and 1 deafness alone.

### 3.2 Genetics

Of our entire cohort, 75 patients (93%) had confirmatory genetic testing with 53 of these (65% of total cohort) having heteroplasmy levels available (in serum for all patients). Of those with genetic testing, 84% had the classic MTTL1 m.3243A > G. Additional genetic testing included MTND5 m.13513G > A (3.7%), MTTV 1624C > T (1.2%), MTND5 m.13045A > C (1.2%), MTTL1 m. 3260A > G (1.2%), MTTL1 m.3251A > G (1.2%). Heteroplasmy levels were obtained in serum with a mean level of 31.6% (range 1–98%). Several patients had additional heteroplasmy levels in muscle or other tissue samples, however, this was not consistently obtained in the vast majority and was not used in comparison.

Of the 42 MELAS patients, 4 patients were diagnosed by biopsy (2 brain, 2 muscle). Two were diagnosed by clinical criteria but did not have genetic or pathologic testing (but did have diagnosis in related family members). In the 30 symptomatic non-MELAS patients, all except 2 had confirmatory genetic testing with the classic MTTL1 m.3242A > G mutation; of the two who did not, one had m.13513G > A in ND5 gene and had MIDD phenotype and the other had biopsy confirmation and had encephalopathy without SLE. All 9 patients in the asymptomatic subgroup had classic MTTL1 m.3243A > G.

### 3.3 MELAS vs. symptomatic non-MELAS vs. asymptomatic patients

**Figure 2:**
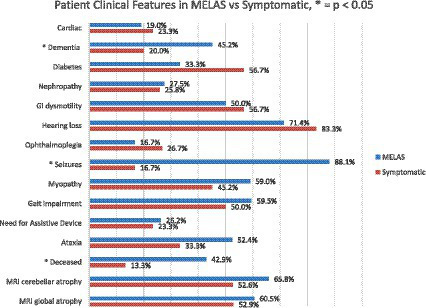
Comparison of clinical features of MELAS vs. symptomatic non-MELAS patients at any point during disease course. Significant differences (*p* < 0.050 are identified by *).

**Figure 3:**
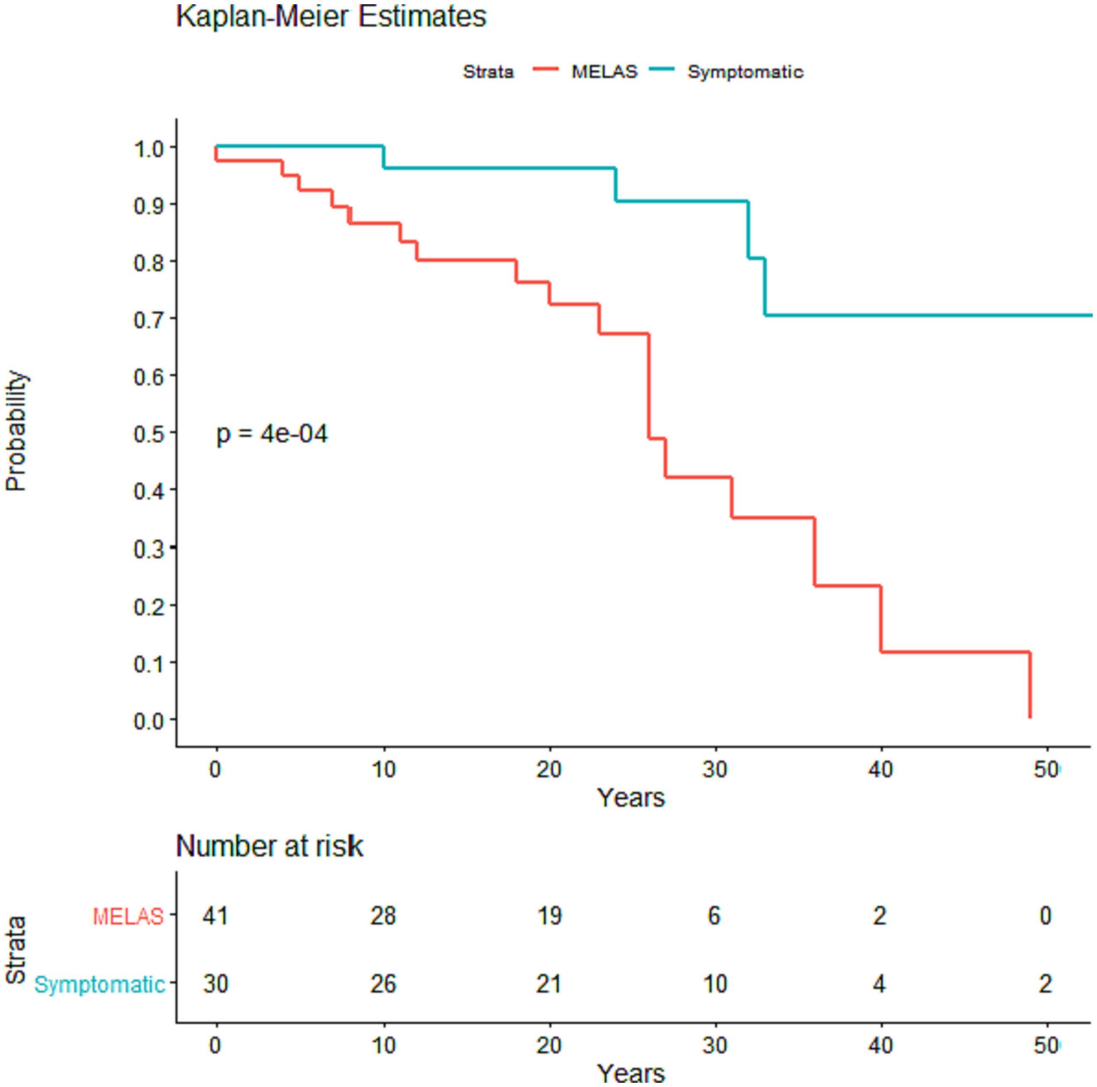
Kaplan–Meier survival curve comparing time from first symptom onset to death in MELAS and symptomatic non-MELAS patients. *P* value was calculated using log rank sum with significance <0.05.

There was no difference in the age at earliest symptom onset between MELAS and symptomatic non-MELAS patients (20.3 vs. 21.5 years) or in follow-up period (5.3 vs. 5.9 years) (Table 1). There was a trend toward increased heteroplasmy levels in MELAS vs. symptomatic non-MELAS patients (39.3 vs. 29.3, respectively) (Table 1). There was a similar trend toward increased baseline lactate in MELAS patients (mean serum lactate 3.4 vs. 2.5 mmol/L, respectively, *p* = 0.06). There was significantly lower BMI seen at initial presentation in patients with MELAS compared to symptomatic non-MELAS patients (mean 18.1 vs. 21.4, *p* = 0.0013). Symptomatic non-MELAS patients were more likely than MELAS patients to present with sensorineural hearing loss as their first symptom (53.3% vs. 21.4%, respectively, *p* = 0.0065) (Table 2). The first presenting symptom in MELAS patients was SLE for 3 patients, cardiac disease for 2 patients, dementia for 1 patient, and headaches for 1 patient. These symptoms were not seen at onset for symptomatic non-MELAS patients, however, the numbers were too small to draw statistical comparisons (Table 2). Throughout their disease course, MELAS patients had higher rates of dementia (45.2% vs. 20.0%, respectively, *p* = 0.0436), seizures (88.1% vs. 16.0%, respectively, *p* < 0.001), and increased mortality (42.9% vs. 13.3%, respectively, *p* = 0.0094) (Figure 2). There was a trend toward higher rates of diabetes in symptomatic non-MELAS patients compared to MELAS (56.7% vs. 33.3, *p* = 0.058). Of note, there were similar rates of gait disturbance and need for assistive devices in both groups. Kaplan–Meier survival curve demonstrated significantly higher probability of longer survival in symptomatic non-MELAS patients compared to MELAS (Figure 3). In grouping symptoms by organ system, MELAS patients had higher rates of neurologic disease than symptomatic non-MELAS patients (100% vs. 64.5%, *p* < 0.001), likely reflecting the increased burden of both SLE and seizures in this cohort (Table 2).

**Table 1:** Comparison of MELAS, symptomatic non-MELAS patients, and asymptomatic patients clinical and laboratory features.

	Asymptomatic (*N* = 9)	MELAS (*N* = 42)	Symptomatic (*N* = 30)	Total (*N* = 81)	*p* value
Duration of follow up (yrs)					
N	9	42	30	81	0.117
Mean (SD)	1.7 (2.4)	5.3 (5.7)	5.9 (5.4)		
Median	1	3	4		
Q1, Q3					
Range	(0–8)	(0–22)	(0–23)		
Age at last follow up					
N	9	42	30	81	*0.0037
Mean (SD)	26.1 (14.9)	37.4 (16.6)	46.1 (15.6)	39.4 (17.0)	
Median	27	38	47.5	42	
Q1, Q3	17, 38	24.2, 48.8	41, 57	26, 52	
Range	(2.3–44)	(7.8–71)	(6–67)	(2.3–53)	
BMI 1st visit					
N	8	37	30	75	*0.0013
Mean (SD)	25.1 (9.3)	18.6 (3.2)	22.0 (5.4)	20.7 (5.4)	
Median	21.2	18.1	21.4	19.7	
Q1, Q3	19.7, 27.4	16.3, 20.8	17.7, 24.3	17.5, 23.1	
Range	(18–46.1)	(13–24.9)	(14.5, 39.5)	(13–46.1)	
Age at earliest symptom					
N		42	30	72	0.781
Mean (SD)		20.3 (14.0)	21.5 (15.4)	20.7 (14.6)	
Median		18.5	20.5	19.5	
Q1, Q3		9.5, 30.8	10.0, 34.8	8.5, 34.0	
Range		(0–53)	(0–55)	(0–55)	
Age at diagnosis					
N	9	42	30	79	*0.0039
Mean (SD)	21.2 (15.1)	33.0 (16.5)	41.4 (15.9)	35.5 (16.7)	
Median	17.0	32.0	43.0	37.0	
Q1, Q3	10, 35.0	21.0, 44.8	32.8, 52.8	21.0, 46.0	
Range	(0.2–43)	(0.6–70)	(2.0–65)	(0.2–70)	
Diagnosed in relative first	9 (100%)	6 (14.3%)	17 (58.6%)	32 (39.5%)	*<0.001
Age at death					
N	0	18	4	22	0.672
Mean (SD)		39.2 (19.5)	45.2 (21.2)	42.0 (18.8)	
Median		40.5	51.0	42.0	
Q1, Q3		24.3, 54.8	37.8, 58.8	24.3, 56.8	
Range		(3–71)	(16–64)	(3–71)	

Age at diagnosis was defined by confirmatory genetic and/or pathologic testing. Heteroplasmy levels are from serum samples. Significant results identified by *.

**Table 2:** Comparison of clinical symptoms and organ systems involved in MELAS and symptomatic non-MELAS patients.

	Asymptomatic (*N* = 9)	MELAS (*N* = 42)	Symptomatic (*N* = 30)	*P* value	Standard onset (*N* = 29)	Late onset (*N* = 13)	*P* value
Gender							
Female	7 (77.8%)	25 (59.5%)	18 (60.0%)	0.58	15 (51.7%)	10 (76.9%)	0.179
Male	2 (22.2%)	17 (40.5%)	12 (40.0%)		14 (48.3%)	3 (23.1%)	
First symptom							
SLE		3 (7.1%)	0 (0.0%)	0.26	2 (6.9%)	1 (7.7%)	1.0
Cardiac		2 (4.8%)	0 (0.0%)	0.51	2 (6.9%)	0 (0%)	1.0
Dementia		1 (2.4%)	0 (0.0%)	1.0	1 (3.4%)	0 (0%)	1.0
Developmental delay		4 (9.5%)	2 (6.7%)	1.0	4 (13.8%)	0 (0%)	0.29
Diabetes		4 (9.5%)	4 (13.3%)	0.71	1 (3.4%)	3 (23.1%)	0.081
Gastric dysmotility		5 (11.9%)	1 (3.3%)	0.39	3 (10.3%)	2 (10.3%)	0.16
Headaches		2 (4.8%)	0 (0.0%)	1.0	2 (6.9%)	0 (0%)	1.0
Hearing loss		9 (21.4%)	16 (53.3%)	*0.0065	4 (13.8%)	5 (38.5%)	0.107
Ophthalmoplegia		1 (2.4%)	1 (3.3%)	1.0	1 (3.4%)	0 (0%)	1.0
Seizures		8 (19.0%)	2 (6.7%)	0.18	7 (24.1%)	1 (7.7%)	0.39
Weakness		3 (7.1%)	3 (10.0%)	1.0	2 (6.9%)	1 (7.7%)	1.0
First organ system							
Neurologic		17 (40.4%)	6 (19.4%)	0.12	15 (51.7%)	2 (15.4%)	*0.0414
Musculoskeletal		9 (22.0%)	5 (16.1%)	0.77	6 (20.7%)	3 (23.1%)	1.0
Endocrine		4 (9.8%)	4 (12.9%)	0.71	1 (3.4%)	3 (23.1%)	0.081
Cardiac		2 (4.9%)	0 (0%)	0.51	2 (6.9%)	0 (0%)	1.0
Renal		0 (0%)	0 (0%)	1.0	0 (0%)	0 (0%)	1.0
Acoustic		10 (24.4%)	16 (51.6%)	0.014	5 (17.2%)	5 (38.5%)	0.24
Organ systems involved							
Neurologic		42 (100%)	20 (64.5%)	* < 0.001	29 (100%)	13 (100%)	n/a
Musculoskeletal		30 (71.4%)	25 (80.6%)	0.42	20 (69.0%)	10 (76.9%)	0.72
Endocrine		13 (31.7%)	17 (54.8%)	0.058	4 (13.8%)	9 (69.2%)	*0.0007
Cardiac		8 (19.5%)	(19.4%)	1.0	5 (17.2%)	3 (23.1%)	0.69
Renal		10 (24.4%)	7 (22.6%)	1.0	3 (10.3%)	7 (53.8%)	*0.0046
Acoustic		30 (71.4%)	26 (83.9%)	0.27	18 (62.1%)	12 (92.3%)	0.668
Number of systems total							
Mean (SD)		3.2 (1.3)	3.3 (1.5)	0.76	2.7 (1.0)	4.1 (1.3)	* < 0.001
Median		3	3		3	4	
Q1, Q3		2, 4	2, 4		2, 3	4, 5	
Range		1–6	1–6		1–6	1–6	

First symptoms are compared between MELAS and symptomatic non-MELAS patient as well as standard onset MELAS (presenting with first stroke like episode at age 40 or younger) vs. late onset MELAS (>age 40). Symptoms are then grouped by organ system (note, GI dysmotility is grouped as musculoskeletal, as this is thought to be due to smooth muscle dysfunction). Significant results are identified by *.

### 3.4 Late vs. standard onset MELAS

There were 29 patients with standard onset MELAS (first SLE < 40 years old) and 13 patients with late onset MELAS (SLE at 40 years old or later). There was no difference in BMI at first presentation between these two groups (Table 3). There was also no difference in baseline or peak lactate during an SLE (Table 3). There was a trend toward increased heteroplasmy in standard onset MELAS compared to late onset, but this was not significant (44.8 vs. 25.3, *p* = 0.176).

**Table 3:** Late onset vs. standard onset MELAS.

	Standard (*N* = 29)	Late onset (*N* = 13)	*p* value
BMI first visit			
N	24	13	0.594
Mean (SD)	18.4 (3.2)	19.0 (3.1)	
Median	17.7	18.8	
Q1, Q3	16.0, 20.9	17.5–20.1	
Range	(14.2–24.8)	(13–24.9)	
Heteroplasmy (%)			
N	15	6	0.176
Mean (SD)	44.8 (30.1)	25.3 (24.6)	
Median	42	17.5	
Q1, Q3	18.5, 58.2	11.0, 26.3	
Range	(5–98)	(6–73)	
Age at earliest symptom			
N	29	13	* < 0.001
Mean (SD)	14.2 (10.8)	33.8 (11.0)	
Median	13	34	
Q1, Q3	5, 20	30.0, 42.0	
Range	(0–36)	(16–53)	
Age at diagnosis			
N	29	13	* < 0.001
Mean (SD)	24.2 (10.4)	52.5 (9.0)	
Median	25	52	
Q1, Q3	20, 32	46, 59	
Range	(0.6–39)	(40–70)	
Time from first symptom to SLE			
N	29	13	0.0156
Mean (SD)	9.3 (9.1)	16.8 (8.3)	
Median	6	18	
Q1, Q3	2, 13	11, 22	
Range	(0–29)	(0–32)	
Time from SLE to death			
N	12	6	0.325
Mean (SD)	6.2 (6.0)	9.3 (6.7)	
Median	4	9.5	
Q1, Q3	2, 7.8	4.3, 14	
Range	(0–20)	(0–18)	
Mean baseline lactate			
N	22	11	0.602
Mean (SD)	3.5 (2.0)	3.1 (1.8)	
Median	3.1	2.9	
Q1, Q3	2.2, 4.0	2.1, 3.9	
Range	(0.9, 8.9)	(0.9–6.9)	
Peak lactate during SLE					Standard (*N* = 29)	Late onset (*N* = 13)	*p* value
N	10	5	0.211
Mean (SD)	5.7 (2.5)	8.2 (5.1)	
Median	5.1	6.7	
Q1, Q3	4.3, 6.2	4.2, 9.5	
Range	(3.0–12.0)	(4.2–16.4)	
Age at death			
N	12	6	* < 0.001
Mean (SD)	28.1 (12.3)	61.5 (8.2)	
Median	26	60	
Q1, Q3	23.3, 40.3	56.5, 68.8	
Range	(3–44)	(51–71)	
Age at insulin dependence			
N	3	6	*0.0226
Mean (SD)	30.7 (3.2)	45.8	
Median	32	47	
Q1, Q3	29.5	45.5, 50.8	
Range	(27–33)	(30–54)	

Comparison of standard onset MELAS (presenting with first stroke like episode at age 40 or younger) vs. late onset MELAS (>age 40) clinical and laboratory features. Age at diagnosis was defined by confirmatory genetic and/or pathologic testing. Heteroplasmy levels are from serum samples. Significant results (*p* < 0.05) identified by *.

**Figure 4:**
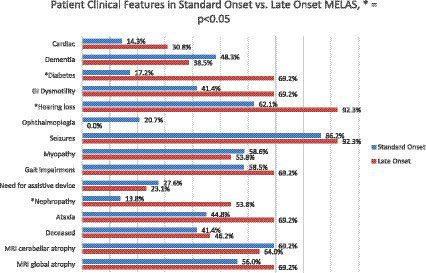
Comparison of clinical features of standard onset MELAS (presenting with first stroke like episode at age 40 or younger) vs. late onset MELAS (>age 40) patients at any point during disease course. Significant differences (*p* < 0.050 are identified by *).

**Figure 5:**
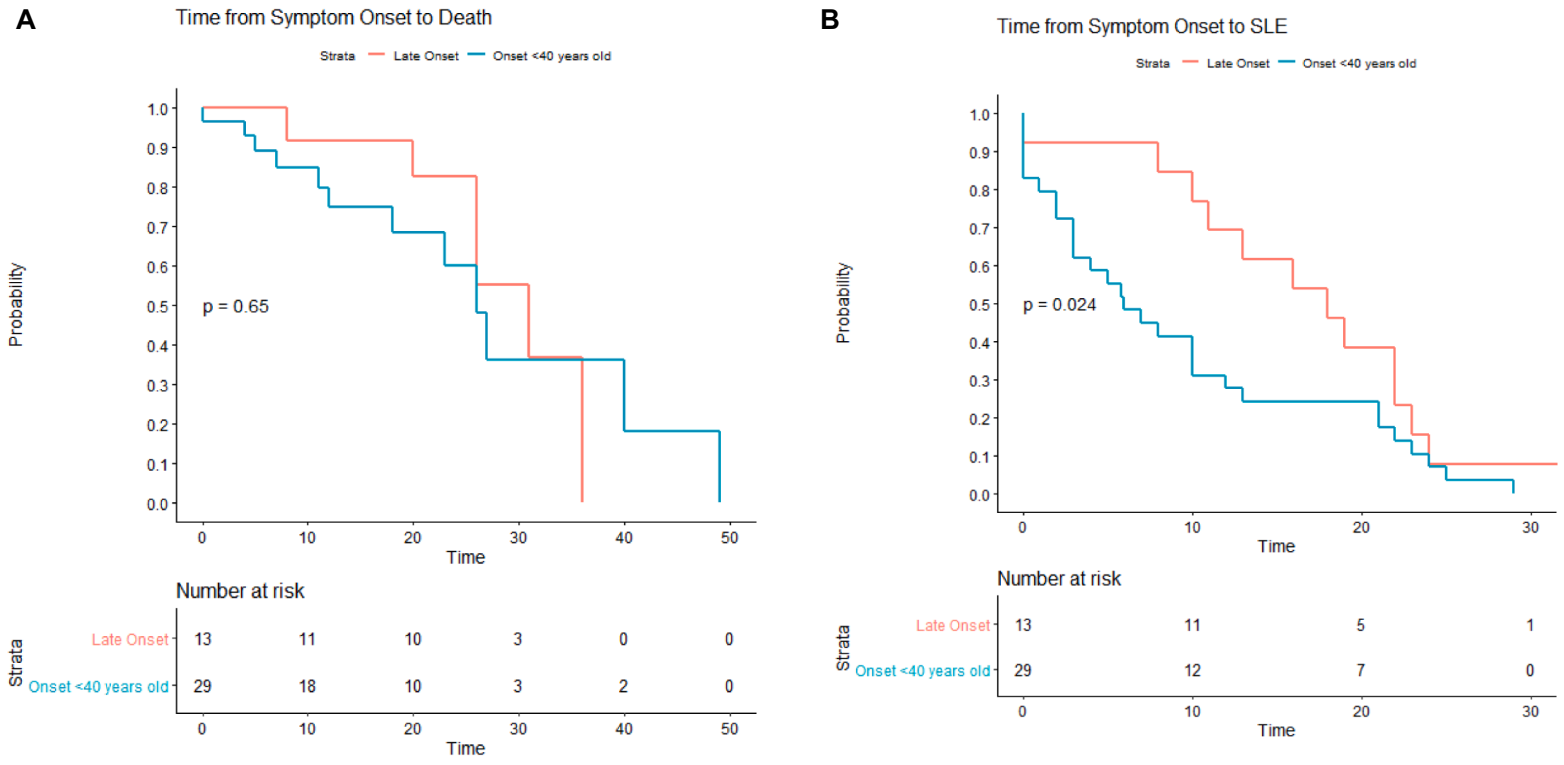
Kaplan–Meier survival curves comparing standard onset MELAS (presenting with first stroke like episode at age 40 or younger) vs. late onset MELAS (>age 40). Comparisons were made in time from symptom onset to death **(A)** and from symptom onset to SLE **(B)**.

In comparing organ systems involved at disease presentation, standard onset patients had a higher rate of initial neurologic symptoms compared to late onset (51.7% vs. 15.4%, *p* = 0.041) (Table 2). There was a trend toward more patients with late onset MELAS presenting with diabetes compared to those with standard onset (23.1% vs. 3.4%, *p* = 0.081) and with hearing loss (38.5% vs. 13.8%, respectively, *p* = 0.107). Throughout their disease course, patients with late onset MELAS had higher rates of diabetes (69.2% vs. 17.2%, respectively, *p* = 0.0032) and nephropathy (53.8% vs. 13.8%, *p* = 0.019) (Figure 4). There was a trend toward higher rates of hearing loss in late onset MELAS (92.30% vs. 62.1%, *p* = 0.067). Of the patients who died, standard onset MELAS had a younger age at death compared to late onset (28.1 vs. 61.5 years, *p* < 0.001) (Table 3), however, Kaplan–Meier curves demonstrated no significant difference between the two groups in time from symptom onset to death (Figure 5A). There was significantly longer time from symptom onset to first SLE in late onset MELAS compared to standard onset MELAS (mean time 16.8 vs. 9.3 years) (Table 3 and Figure 5B).

## 4 Discussion

Our study supports the notion that MELAS exists on a spectrum with some patients presenting in early life (adolescence to young adulthood), but with many patients presenting later. In addition, our cohort demonstrates that the initial symptoms often precede the first SLE by years to decades. Indeed, we also observed that symptomatic non-MELAS patients had a similar age of onset and a similar number of organ systems affected as MELAS patients, despite not having had an SLE. As our study was a retrospective cohort study and patients were not followed to the end of life, it is possible that some of these symptomatic non-MELAS patients may have gone on to develop SLE later in life. Indeed, the late onset MELAS group highlights that many patients look phenotypically similar to the symptomatic non-MELAS group until the presentation of SLE.

Our study did observe differences in BMI between these subgroups at first presentation. Patients with MELAS had significantly lower BMI at presentation compared to symptomatic non-MELAS and asymptomatic patients. This is likely due to more severe myopathy in MELAS patients, as a recent study highlighted axial skeletal muscle loss corresponds with disease severity in MELAS (19). Gastric dysmotility could also be a contributing factor. While that study found axial muscle mass to be a more sensitive biomarker of disease severity than BMI, this may be more difficult to apply to clinical practice and our study highlights that BMI may still be a useful metric in the initial evaluation of these patients. Symptomatic non-MELAS patients were more likely to have diabetes mellitus and hearing loss than MELAS patients and were more likely to present with hearing loss as their initial symptom, which may reflect the MIDD subtype of that cohort. Conversely, MELAS patients were more likely to suffer neurologic symptoms (excluding SLE), with seizures and dementia being more common. Of note, we did not specifically include psychiatric manifestations, which can often be a significant comorbidity in these patients (20, 21). We observed higher mortality in MELAS patients compared to symptomatic non-MELAS patients and although there were no differences in age at symptom onset, patients with MELAS had a lower survival probability from symptom onset than symptomatic non-MELAS.

The MELAS cohort demonstrated variability in age of first SLE; while many presented in young adulthood, roughly 1/3 (13 patients) presented after the age of 40. The increased rates of diabetes, deafness, and nephropathy in the late onset cohort and the trend toward increased presentation with diabetes as first symptom suggests that many of these patients may have initially been classified as MIDD but then later developed more fulminant mitochondrial disease with encephalomyopathy and SLE. Given that many patients with MIDD do not go on to develop MELAS, it is worth asking why certain patients would develop MELAS later. The reasons for this are unclear, but may be due to varying heteroplasmy levels in different organ systems or possibly additional genetic or environmental factors affecting different organ systems at different time points. There was also a higher number of organ systems involved in the late onset cohort, which is explained by higher rates of diabetes and nephropathy in this subgroup. It is interesting to note that there was no significant difference between onset of SLE and death in these two groups, whereas there was a significant difference in time from first symptom onset to presentation of SLE. This further suggests that this late onset subset has a longer period of milder symptoms but then eventually follows a similar disease course as standard onset MELAS. Standard onset patients had higher rates of neurologic involvement as the first symptom, which may explain the earlier SLE and faster disease progression in this cohort.

The reasons for this variability in presentation of SLE are not clear from our data. While there was a trend toward increased serum heteroplasmy in patients with earlier presentation and in MELAS compared to symptomatic non-MELAS patients, this was not significant in our cohort. It is possible that a larger sample size or a population-based sample would have been powered enough to detect a significant difference, as has been previously described (15). The testing in our cohort was limited to serum samples, which is commonly utilized in clinical practice, and yet varying heteroplasmy in different tissue types could be an explanation for differing organ system involvement or age at presentation; CSF heteroplasmy levels are not routinely tested, however, the earlier presentation of neurologic symptoms and earlier SLE seen in the standard onset group could be due to a higher heteroplasmy levels in the brain. Further studies looking at CSF heteroplasmy levels would be useful in answering this question.

Despite the differences between symptomatic non-MELAS patients and MELAS patients, and between standard onset MELAS and late onset, it remains difficult to distinguish these trajectories clinically. There remains a need to accurately predict which patients will develop earlier or more severe disease course and while our study sheds a small amount of light on this, additional biomarkers are needed to help guide clinical counseling and treatment options.

There are several limitations to our study. The retrospective nature of our cohort study has the potential for recall bias. Most patients had relatively short follow up compared to their overall disease course and there was considerable variability in follow up frequency among patients. Our cohort also likely suffers from some degree of selection bias in that patients with more severe disease are more likely to present to a quaternary referral center for evaluation. A larger population-based study such as those in Finland (22) and Japan (23) would be needed to really understand the incidence of asymptomatic carriers and would likely help flesh out differences in disease presentations in those with milder symptoms. While we did include a small group of asymptomatic carriers, there was selection bias in that these patients all had family members who were symptomatic. There was also relatively short follow up for this subgroup, and so it is difficult to know if and when they may have developed symptoms in the future. In addition, the fact that 20 patients were related in 8 families could also introduce other genetic factors that were not included in mitochondrial DNA testing.

In summary, our study demonstrates the heterogeneity of MELAS. There are many patients with similar genetic mutations as MELAS who are either asymptomatic or have less severe mitochondrial disease, and some patients who do not develop full MELAS with SLE until much later in life. Patients with MELAS are more likely to present with a lower BMI and to have seizures or other neurological manifestations in their disease course and are less likely to present with hearing loss, compared with symptomatic non-MELAS patients. Patients with late onset MELAS are more likely to have diabetes, hearing loss, and nephropathy whereas patients with standard onset MELAS are more likely to present with neurologic symptoms. Despite differences in initial onset of SLE, both cohorts have similar survival after the onset of first SLE, highlighting the overall severity and mortality of MELAS once patients present with SLE.

## Data availability statement

The raw data supporting the conclusions of this article will be made available by the authors, without undue reservation.

## Ethics statement

The studies involving humans were approved by Mayo Clinic Institutional Review Board. The studies were conducted in accordance with the local legislation and institutional requirements. The participants provided their written informed consent to participate in this study.

## Author contributions

BC: Conceptualization, Investigation, Methodology, Writing – original draft. JP: Investigation, Writing – review & editing. JM: Formal analysis, Writing – review & editing. RG: Conceptualization, Investigation, Methodology, Project administration, Writing – review & editing.
